# Epigenetically regulated Fibronectin leucine rich transmembrane protein 2 (FLRT2) shows tumor suppressor activity in breast cancer cells

**DOI:** 10.1038/s41598-017-00424-0

**Published:** 2017-03-21

**Authors:** Hansol Bae, Byungtak Kim, Hyunkyung Lee, Seungyeon Lee, Han-Sung Kang, Sun Jung Kim

**Affiliations:** 1 Department of Life Science, Dongguk University-Seoul, Goyang, Republic of Korea; 2 Research Institute and Hospital, National Cancer Center, Goyang, Republic of Korea

## Abstract

To identify dysregulated genes by abnormal methylation and expression in breast cancer, we genome-wide analyzed methylation and expression microarray data from the Gene Expression Omnibus and the Cancer Genome Atlas database. One of the genes screened *in silico*, FLRT2, showed hypermethylation and downregulation in the cancer dataset and the association was verified both in cultured cell lines and cancer patients’ tissue. To investigate the role of FLRT2 in breast cancer, its expression was knocked down and upregulated in mammary cell lines, and the effect was examined through three levels of approach: pathway analysis; cell activities such as proliferation, colony formation, migration, and adhesion; target gene expression. The top pathway was “Cellular growth and proliferation”, or “Cancer”-related function, with the majority of the genes deregulated in a direction pointing to FLRT2 as a potential tumor suppressor. Concordantly, downregulation of FLRT2 increased cell proliferation and cell migration, while overexpression of FLRT2 had the opposite effect. Notably, cell adhesion was significantly decreased by FLRT2 in the collagen I-coated plate. Taken together, our results provide insights into the role of FLRT2 as a novel tumor suppressor in the breast, which is inactivated by hypermethylation during tumor development.

## Introduction

Methylation of CpG sites in DNA is a key epigenetic mechanism in transcriptional regulation, including gene imprinting and silencing of coding genes as well as repetitive DNA elements^1^. One of the varied functions of methylation is to regulate gene expression and this has been widely studied in relation to cancer, especially with a view to elucidating the effect of methylation on carcinogenesis and prognosis^2^. The impact of methylation is even greater in relation to tumor suppressor genes^3^ or oncogenes^4^. Hypermethylation of tumor suppressor genes or hypomethylation of oncogenes can suppress or activate the corresponding genes, and these processes have been identified as cancer-causing events^5, 6^.

GSTP1 is a well-known tumor suppressor that carries hypermethylation on promoter CpGs in various cancer types^7^. Abnormally high methylation inactivates GSTP1 and is associated with reduced cell detoxification and anti-cancer enzyme activity^8^. Hypermethylation is pronounced in cancer, but not in benign processes^9^, and this makes the gene distinctive as an epigenetic cancer marker. To cite an example of an oncogene, MET has been identified as a methylation marker that is hypomethylated in several types of cancer, including pancreatic, liver, and breast cancer, and associated with poor prognosis for patients^10, 11^.

The mining of epigenetically regulated tumor suppressors and oncogenes is directly connected to establishing biomarkers that could eventually become useful parameters in predicting clinical outcomes or in prognostication^12^. For instance, the methylation levels of well-known tumor suppressors such as RASSF1A, ADAM33, and NEFL show a specific methylation range for specific cancer stages and gradually increase following the development of cancer^13–15^.

FLRT2 (Fibronectin leucine rich transmembrane protein 2) is a member of the FLRT family proteins, which contain 10 LRR (leucine-rich repeats) domains and a transmembrane domain^16^. FLRT proteins are known to interact with several other proteins, such as ROBO1^17^, LPHN3 and UNC5^18, 19^. Through these interactions, FLRTs function as ribonuclease inhibitors^20^, virulence factors^21^, or splicing mediators^22^. During development, all FLRTs are strongly expressed in a subset of sclerotome cells and interact with FGFR1 in the FGF (fibroblast growth factor) signaling pathway^23^. In addition to this function in development, FLRT2 acts as an adhesion molecule by interacting with fibronectin^24^ in either a repulsive or adhesive manner. This suggests a possible relationship between FLRT2 and cancer metastasis.

The aim of this study was to develop methylation markers through *in silico* meta-data analysis, and this revealed FLRT2 to be hypermethylated in breast cancer. The relationship between methylation and expression was addressed by examining the expression of the gene in breast cancer cell lines as well as tissues. To characterize the role of FLRT2 in tumorigenesis, downregulation and upregulation were induced in mammary cell lines, and the effects on cell proliferation, migration, and apoptosis were examined. The results suggest that FLRT2 may have tumor suppressor activity in breast cancer.

## Results

### FLRT2 is hypermethylated and downregulated in breast cancer

To identify novel genes that play a key role in breast cancer development, such as being deregulated due to aberrant promoter methylation, genome-wide methylation data deposited in GEO were analyzed. Methylation assay data were obtained by means of an Illumina Infinium HumanMethylation27 BeadChip covering 27,578 CpG sites in the promoter regions of 14,495 genes. The array results included data extracted from GSE32393 data set that contained 23 normal and 114 cancer tissue array data. Two or three cancer arrays were removed due to lack of grade or ER status. Ten more normal tissue data were added from the GSE33065 and GSE20713 set to complement the less number of normal tissue array (Supplementary Table S1). Genes that did not exhibit statistically significant (p > 0.05) methylation differences were excluded in order to identify genes that are aberrantly methylated in cancer. Thirty genes that displayed differential methylation (Δβ), with at least a two-fold difference between normal and cancer tissue, were finally identified.

Next, a pool of deregulated genes was assembled by comparing expression array data from normal cell line MCF-10A and breast cancer cell lines MDA-MB-231, T-47D, and MCF-7 (Supplementary Table S2). This yielded 13 genes that satisfied the screening criteria (p ≤ 0.05, Δβ ≥ 2.0, and |Δexpression| ≥ 1.5) (Table 1). Of the 13 genes, BNC1 and FBN2 were already known to be deregulated as a consequence of aberrant promoter methylation in breast cancer and a number of other cancers^25, 26^. This pilot study repeatedly displayed hypermethylation and downregulation patterns of FLRT2 in breast cancer cells *in silico*.

**Table 1:** Selected genes that show hypermethylation and downregulation in breast cancer cells.

Gene	cg ID	Brief function	Δβ-value^a^	Expression fold change^b^
SFRP1	cg22418909	Stress-associated endoplasmic reticulum protein	0.25	−2.2
BNC1	cg18952647	Zinc finger transcription factor	0.33	−3.0
CCND2	cg12385902	Regulators of cyclin-dependent kinases	0.23	−1.6
GPM6B	cg21229055	Involved in cellular housekeeping functions	0.22	−1.7
FLRT2	cg17410236	Function in cell adhesion and/or receptor signalling	0.32	−1.5
FBN2	cg27223047	Play a role in early elastogenesis	0.27	−1.9
BCAN	cg21475402	Formation of the brain extracellular matrix	0.26	−1.5
GIPC2	cg24496666	PDZ domains organize and maintain complex scaffolding formations	0.25	−1.6
PLSCR4	cg24315815	Mediate accelerated ATP-independent bidirectional transbilayer migration of phospholipids	0.23	−1.7
LCAT	cg26924825	Remove cholesterol from the blood and tissues	0.23	−1.5
APCDD1	cg19264571	Inhibits Wnt signaling in a cell-autonomous manner	0.22	−1.5
FBN1	cg18671950	Fibrillin-1 attach to each other and to other proteins to form microfibrils	0.22	−1.5
PLD5	cg12613383	Inactive Phosphatidylcholine-Hydrolyzing Phospholipase D5	0.21	−2.3

^a^The values are obtained by subtracting the methylation level in normal cells from that in cancer cells.

^b^The values are obtained by dividing the expression level in cancer cells by that in normal cells.

**Figure 1:**
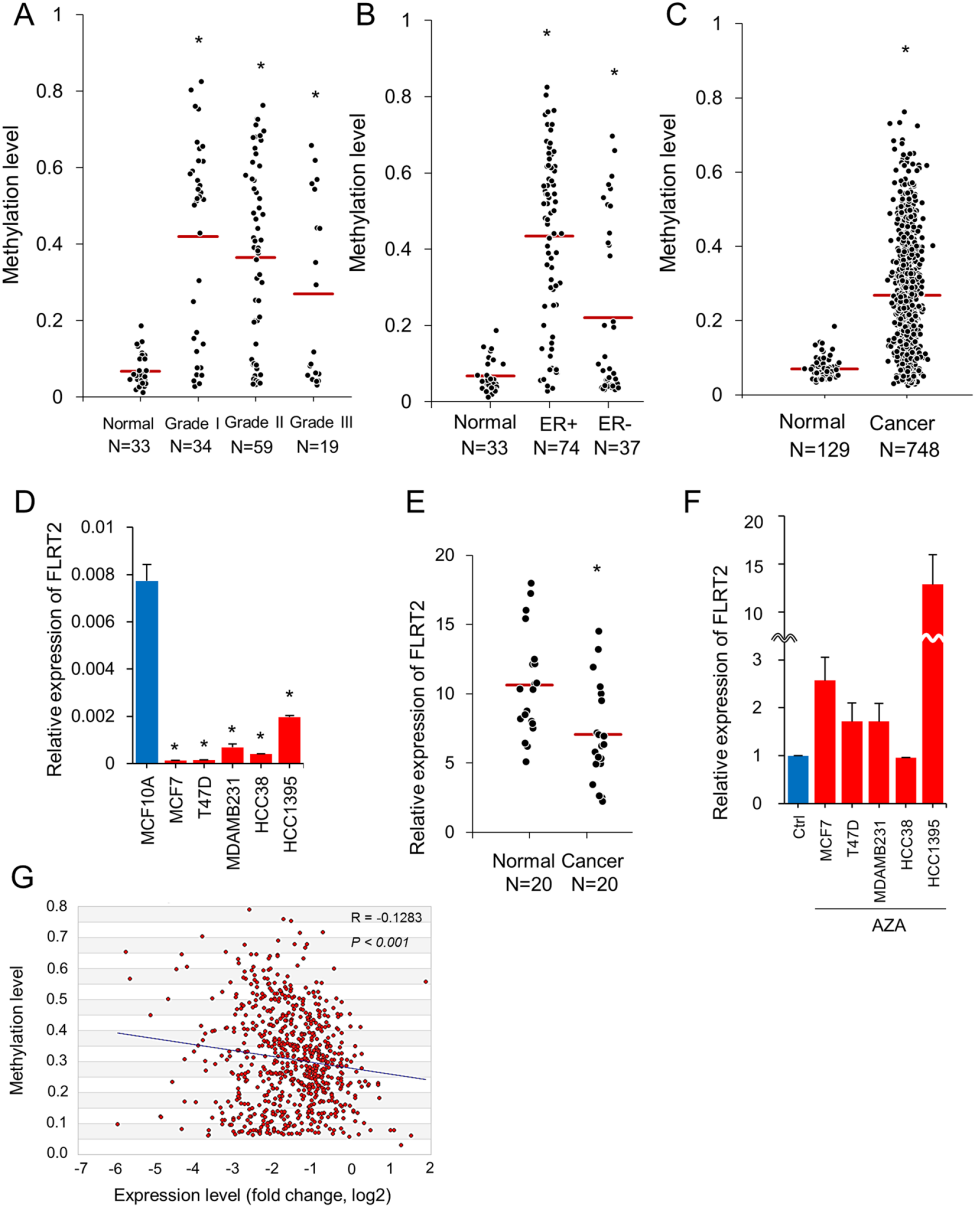
FLRT2 is hypermethylated and downregulated in breast cancer. (**A**) The CpG methylation level of FLRT2 was extracted from the methylation chip data of the GEO (http://www.ncbi.nlm.nih.gov/geo/) (**A**,**B**) and the TCGA database (**C**). The methylation level was stratified at dot plots according to the cancer grade (**A**) and ER status (**B**) or for the whole cancer samples (**C**). The median value is indicated by a red bar. The expression level of FLRT2 was examined in breast cell lines (**D**) and tissues (**E**) by real-time RT-PCR. MCF-10A is a normal cell line while the others are cancer cell lines. (**F**) Demethylation of CpGs was induced by 5-Aza-2′-deoxycytidine (AZA) in cancer cell lines and FLRT2 expression was analyzed by real-time RT-PCR. All RT-PCR experiments were carried out in triplicate and the data are represented as mean ± SD. (**G**) Association of methylation and expression in breast cancer tissue was plotted for 713 samples from the TCGA database (http://cancergenome.nih.gov/) (*P* < 0.001).

**Figure 2:**
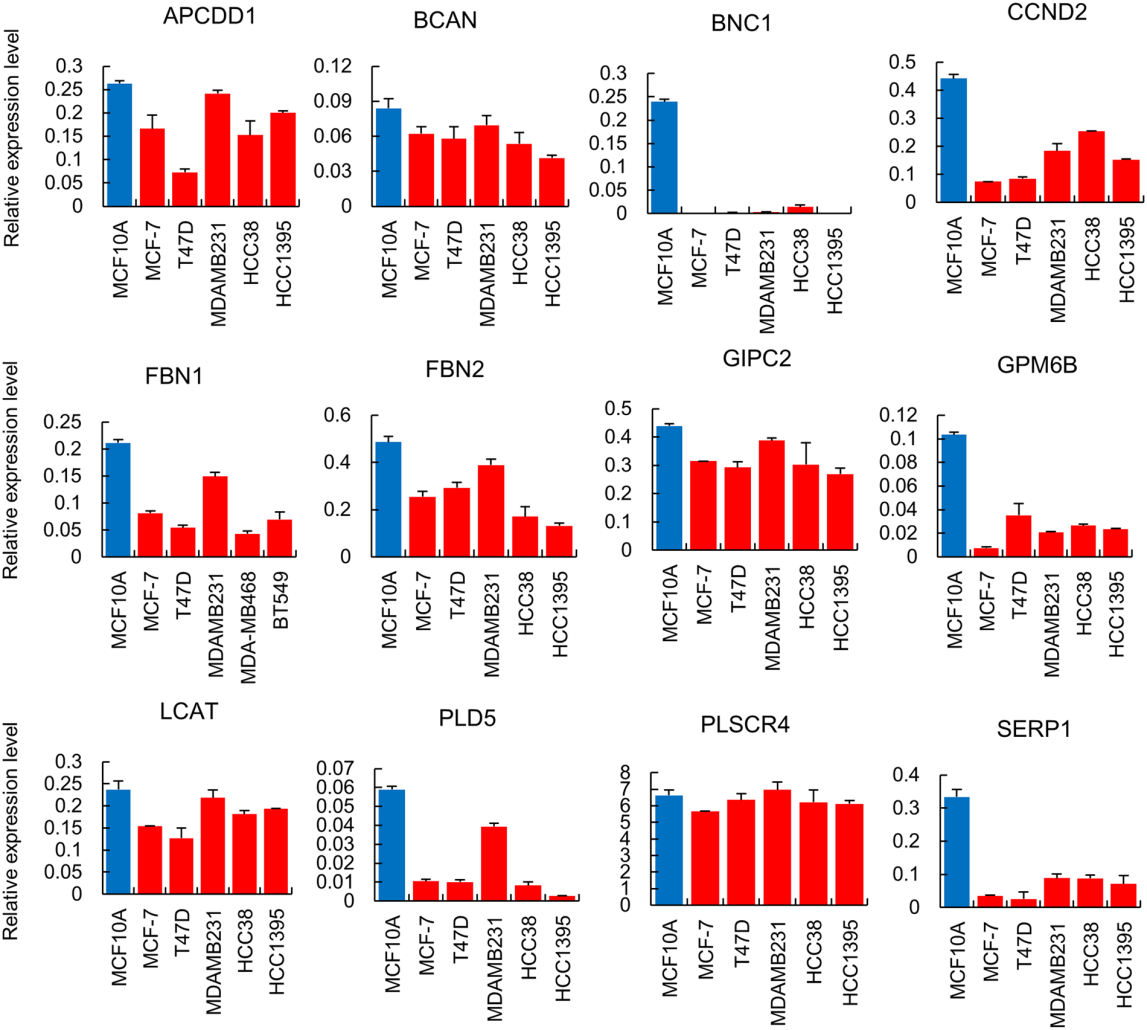
RT-PCR analysis of *in silico* filtered genes. RT-PCR was carried out for 12 genes in normal breast cell line MCF-10A and five breast cancer cell lines, which were filtered from databases by showing a significant methylation and expression change between normal and cancer cells. All reactions were performed three times, and the results are shown as the mean ± SD.

According to the GEO data, promoter methylation of FLRT2 appeared to be higher in cancer of grade I~III than in the normal breast tissue (Fig. 1A). In cancer, ER+ showed higher methylation than ER− (Fig. 1B). The *in silico* analysis was extended to the data from the TCGA database that offered 129 normal and 748 cancer tissues of which grade or ER status was not informed. As like in GEO, the methylation level was higher in cancer patients (Fig. 1C). To validate the *in silico* pattern, the expression level of FLRT2 was measured in breast-derived cell lines and tissues. FLRT2 was found to be downregulated in all examined breast cancer cell lines, MCF-7, T47D, MDA-MB-231, HCC38, and HCC1395, compared to normal cell line MCF-10A (Fig. 1D). All the 12 other genes that have been identified with FLRT2 *in silico* to be hypermethylated in cancer also showed downregulation in MDA-MB-231 compared to in MCF-10A except for PLSCR4 (Fig. 2). Next, FLRT2 expression was examined in 20 pairs of breast cancer tissue and nearby normal tissue. This indicated that the gene was significantly downregulated in cancer tissues compared to normal tissues (p < 0.05) (Fig. 1E and Supplementary Fig. S1). To confirm whether reduced expression of FLRT2 is caused by a high level of methylation, 5-Aza-2′-deoxycytidine was applied and the expression of FLRT2 was measured to gauge whether expression recovers in the absence of hypermethylation. FLRT2 expression increased in all the examined cancer cell lines except for HCC38 following treatment of 5-Aza-2′-deoxycytidine (Fig. 1F).

The association between methylation and expression of FLRT2 was further analyzed using 713 cancer data from the TCGA breast database, which observed a close association (*R* = −0.128, *P* < 0.001) (Fig. 1G). Interrogating the GOBO database revealed that patients with higher expression of FLRT2 were more likely to experience increased distant metastasis-free survival (DMSF), especially in the ER+, luminal A, and lymph node negative tissue (*p* < 0.05) (Supplementary Fig. S2). The combined results of *in silico* and molecular experiments suggest that FLRT2 is potentially an epigenetically modulated tumor suppressor in breast cancer.

### FLRT2 is involved in anti-cancer pathway

**Figure 3:**
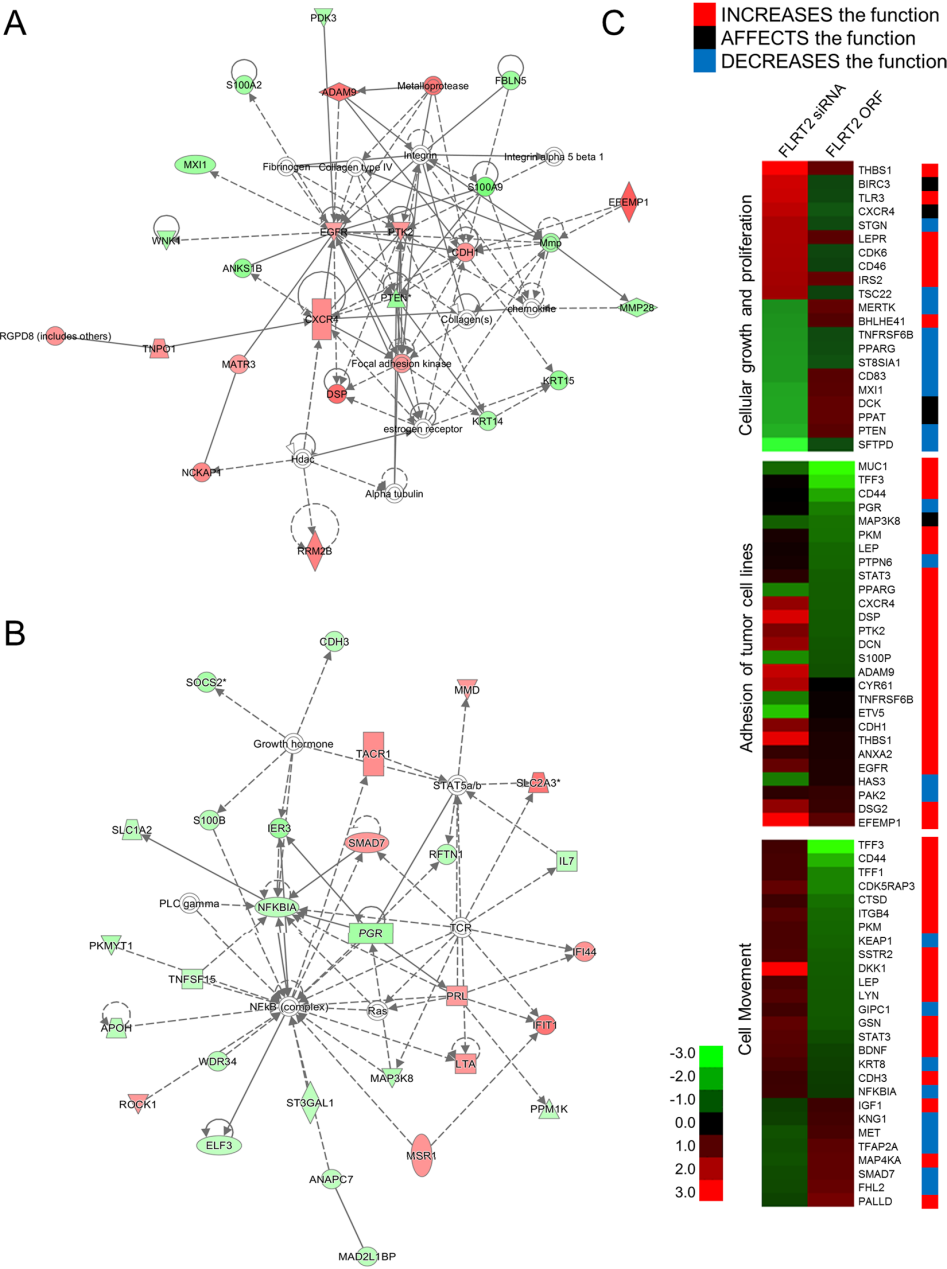
FLRT2 regulates genes as a way of inhibiting cell proliferation. Highest confidence IPA networks of genes displaying altered expression as a consequence of downregulating (**A**) and overexpressing (**B**) FLRT2. The top networks were “Infectious Disease, Auditory Disease, and Cancer” for downregulation (**A**) and “Infectious Disease, Cell Signaling, Cell-To-Cell Signaling, and Interaction” for overexpression (**B**). Upregulated genes are shaded in red, while downregulated genes are shaded in green, with the color intensity indicating the magnitude of the expression change. Solid and dashed lines respectively represent direct and indirect interactions. (**C**) Of the genes affected by FLRT2, genes related to “cellular growth and proliferation”, “adhesion of tumor cell lines”, and “cell movement” were clustered in heatmaps. The colored bar to the right of the gene symbol signifies the activity of the cellular biofunctions based on information from Qiagen (http://www.sabiosciences.com/pathwaysonline/). Genes represented by red and blue bars exhibit expression changes in the direction of an increase and a decrease of the function, respectively. Genes in black bar are to affect the function, i.e., the genes are associated with biological function, but they have no direct effect on the activity.

To verify that FLRT2 is a potential tumor suppressor, genome wide expression was examined by microarray analysis after induction of downregulation and upregulation of FLRT2 by means of an siRNA- and an FLRT2-expressing plasmid vector system, respectively, in cultured mammary cells. Deregulation of FLRT2 was confirmed by qRT-PCR (Supplementary Fig. S3). In siRNA-transfected MCF-10A cells, a total of 296 genes, comprising 155 upregulated and 141 downregulated genes, fitted our criterion of a higher than two-fold expression change and were submitted to IPA. The resulting top network was ‘Cancer, cellular movement, and tumor morphology’ (Fig. 3A). EGFR and focal adhesion kinase (FAK) were at hubs of the network with being upregulated. Recently, EGFR-signaling was revealed to trigger the tyrosine phosphorylation of β4 integrin, which, in turn, recruits FAK^27^.

In case of the FLRT2-overexpressing MCF-7 cells, a total of 92 genes, comprising 31 upregulated and 61 downregulated genes, exhibited a higher than two-fold expression change. The top IPA network from the FLRT2 overexpressing genome was ‘Cellular growth and proliferation, tissue development, and cellular movement’ (Fig. 3B). Of note, NF-κB complex took part in a hub of the network with the majority of genes regulating the complex to be downregulated. Also, many cancer-related genes such as IL7, S100B and IER3 showed an altered expression pattern^28–30^. Based on the knowledge that FLRT2 is responsible for cellular growth and proliferation, cellular adhesion, and movement, the deregulated genes associated with those pathways were arranged in heat maps (Fig. 3C). To identify the coincidence between the gene’s function and direction of expression change by FLRT2 deregulation, an extra bar having the three-color code was added to the right of the heatmap. For example, TLR3 bears cell proliferation-stimulation activity and the expression was changed toward the way of confirming proliferation-suppressive activity of FLRT2. Notably, the expression of proliferation-enhancing genes such as TLR3 and IRS2 was increased when FLRT2 was down-regulated. In contrast, expression of cell proliferation-inhibiting genes such as PTEN and PPARG was decreased when FLRT2 was down-regulated.

**Figure 4:**
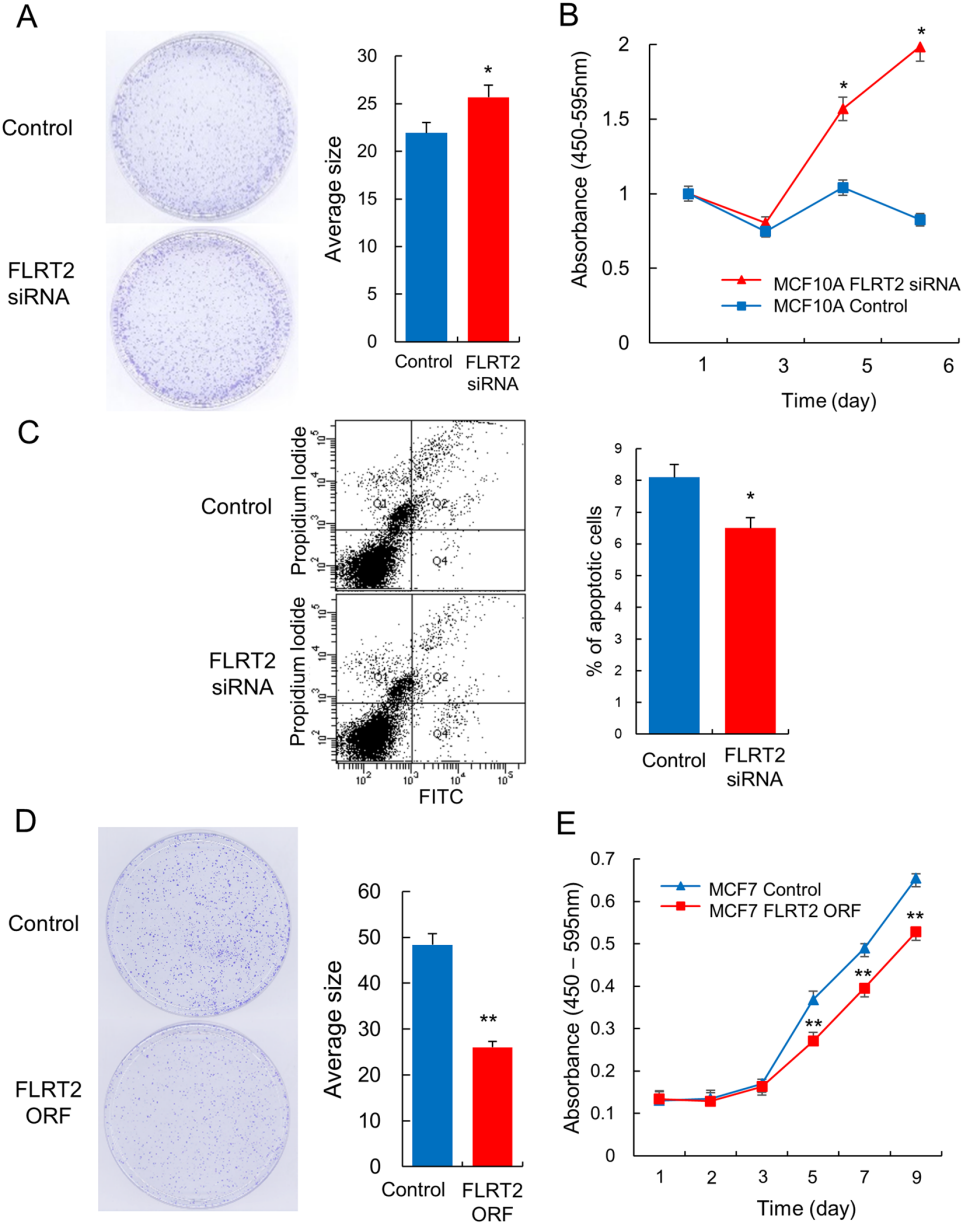
FLRT2 suppresses cell growth but stimulates apoptosis. FLRT2 was downregulated with an siRNA in MCF-10A, normal breast cells and the effect on cell proliferation and apoptosis was examined by colony formation analysis (**A**), CCK-8 assay (**B**), and flow cytometry (**C**). FLRT2 was upregulated with an FLRT2 ORF-containing plasmid expression vector in MCF-7, breast cancer cells and the effect on cell proliferation was examined by colony formation analysis (**D**) and CCK-8 assay (**E**). All experiments were performed at least three times independently, and the results are shown as mean ± SD in the bar graphs. Representative images are shown for colony formation and flow cytometry.

To validate the involvement of FLTR2 in tumor-suppressive activity, colony formation and cell proliferation assays were performed after down- as well as upregulation of the gene in cultured cells. When FLRT2 was downregulated in MCF-10A, a normal mammary cell line, the cells showed increased proliferation judged by colony formation and dye-based cell proliferation assay (Fig. 4A and B). Flow cytometry analysis for FLRT2-downregulated MCF-10A cells revealed a 19% decrease of apoptotic rate, supporting an anti-proliferation role for FLRT2 (Fig. 4C). In contrast, upregulation of FLRT2 in MCF-7, a cancer cell line that expresses FLRT2 at a lower than normal level, showed decreased proliferation, confirming anti-proliferation activity of FLRT2 (Fig. 4D and E). These results are consistent with the results of the IPA pathway analysis in which many genes were deregulated by FLRT2 in the direction of suppressing cancerous characteristics.

### FLRT2 affects cell migration

As shown in Fig. 3C, a set of genes related to movement and adhesion of tumor cell lines were differentially expressed after FLRT2 regulation. The expression of many genes that induce adhesion, such as CXCR4 and DCN, and cellular movement in cancer, such as DKK1 and ITGB4, evidently increased when FLRT2 was downregulated, and decreased when FLRT2 was upregulated. CXCR4 and DCN are known to stimulate cell adhesion in various cell types^31, 32^. DKK1 and ITGB4 are known to promote cell migration and invasion^33, 34^.

**Figure 5:**
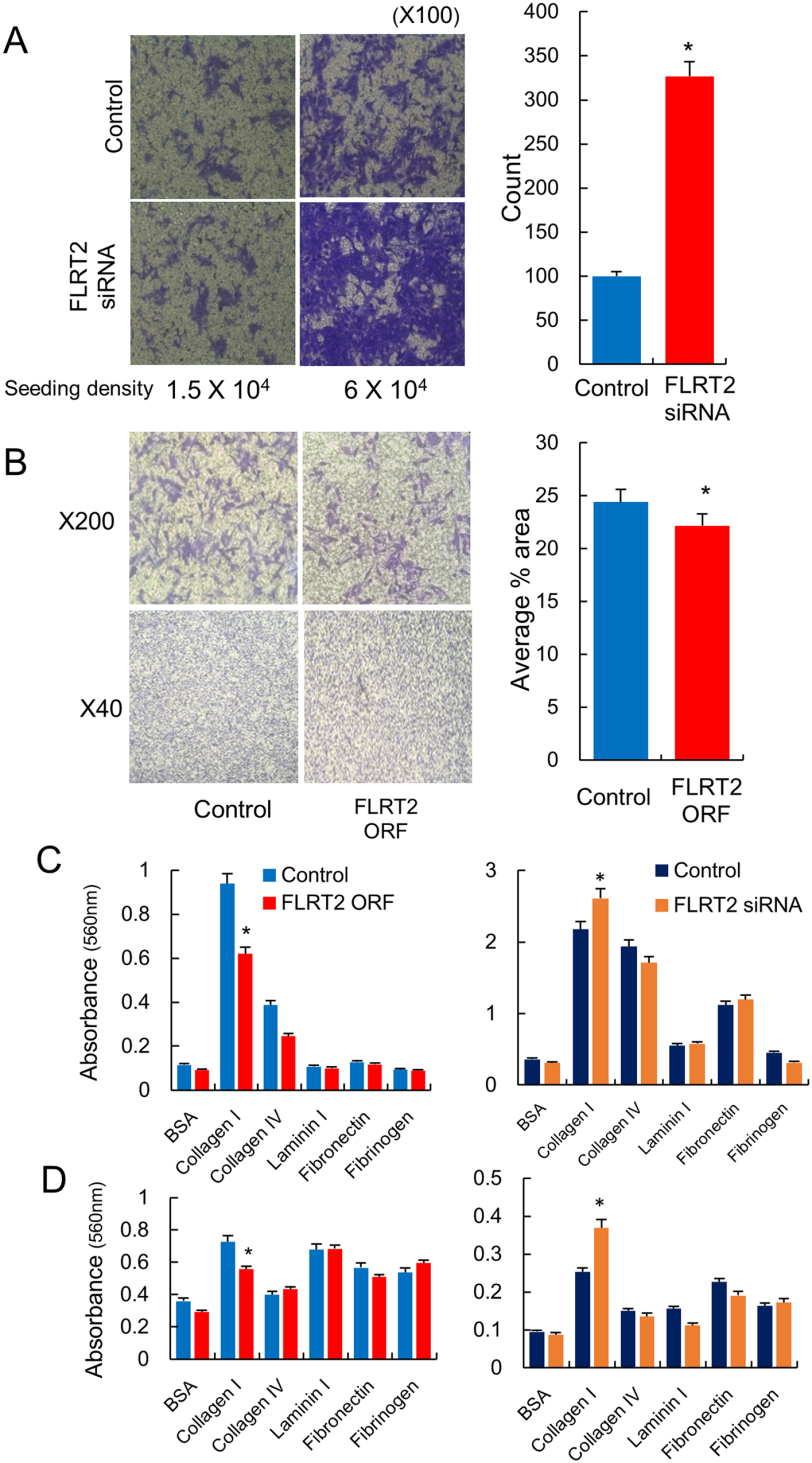
FLRT2 suppresses migration and adhesion. FLRT2 was downregulated with an siRNA in MCF-10A, normal breast cells (**A**) or upregulated in MDA-MB-231, breast cancer cells (**B**) and the effect on cell migration was examined. The images are taken from at least three independent experiments, and the results are shown as means with standard errors in the bar graphs. For adhesion assay, FLRT2 was upregulated or downregulated in MCF-7 (**C**) and MCF-10A (**D**), and the effect on cell adhesion was examined. The results are from three independent experiments and shown as the mean ± SD.

To examine the effects of FLRT2 on cell movement and adhesion, an *in vitro* cell migration assay was carried out after induction of dysregulation of the gene. As a result, cell migration of the FLRT2 siRNA-treated MCF-10A cells increased remarkably, by approximately 3.5-fold (Fig. 5A). To observe the migration of cancer cells that overexpresses FLRT2, MDA-MB-231 cells were chosen instead of MCF-7 because the latter are known to be unsuitable for migration assays owing to their low migratory ability^35^. FLRT2-overexpressing cells showed a slight decrease in migration compared to control cells (Fig. 5B). These results suggest that FLRT2 inhibits cell migration.

To further understand the metastatic function of FLRT2 in correlation to cell adhesion, an adhesion assay for five extracellular matrix proteins was carried out with MCF-7 (Fig. 5C) and MCF-10A (Fig. 5D) cells after deregulation of FLRT2. This showed that cell adhesion significantly decreased when FLRT2 was upregulated in both cells by means of an ORF plasmid vector, but increased when FLRT2 was downregulated with an siRNA in a collagen I-coated plate. Collagen I is a factor that promotes migration in various cancer cells^36^. The observation of decreased migration in FLRT2-overexpressing MDA-MB-231 cells is thought to result from weakened adhesion of the cancer cells with collagen I.

## Discussion

The aim of this study was to develop a breast cancer biomarker that is regulated through epigenetic mechanisms and therefore could be used to diagnose the cancer. To this end, a meta-data analysis of methylation chips was carried out, which produced 13 candidate genes, including FLRT2 (Table 1). So far, little is known about the epigenetic regulation as well as expression of FLRT2 in cancer. For the methylation of promoter CpGs, hypermethylation has recently been reported only in prostate cancer on the basis of a comprehensive high-throughput array-based relative methylation (CHARM) assay^37^. However, neither the methylation in other cancer tissues, including breast cancer, nor the dysregulation of FLRT2 in cancer has been studied. The finding of the hypermethylation for the gene in breast cancer in addition to previously reported prostate cancer prompted us to examine whether the phenomenon was a universal. Searching the public methylation databases obtained with methylation microarrays revealed hypermethylation of FLRT2 at the promoter in other major cancers, including prostate cancer, lung cancer, and breast cancer^37, 38^. The recovery of gene expression in all the breast cancer cells except for HCC38 after induction of demethylation by 5-Aza-2′-deoxycytidine indicates that the methylation change induces downregulation of the gene. HCC38 may have not undergone an appropriate demethylation, or a regulatory mechanism(s) other than methylation may exist. Determination of a specific CpG(s) responsible for the gene expression and its action mechanism should be revealed in further studies. Of note, the IPA network analysis in the normal and cancer cell line where FLRT2 was ectopically dysregulated identified cancer-related pathways in common, suggesting that FLRT2 is closely involved in carcinogenesis. In fact, many of the affected genes by FLRT2 were oncogenes such as TLR3 and IRS2, or tumor suppressors such as PTEN and PPARG.

Structural features of FLRT2 suggest that it functions either as an adhesive or a repulsive signal during organ development. During cortical and vascular development, FLRT2 acts as an adhesion molecule^19^. In contrast, the protein tended to assume a repulsive function during neuronal migration^39^. In this study, up- and downregulation of FLRT2 decreased and increased cell adhesion to a specific matrix protein, collagen I, respectively, which implies that FLRT2 inhibits adhesion to collagen I. This observation is consistent with the change of expression of a set of genes involved in “Adhesion of tumor cell” or “Cell movement”. For example, DSG2 (1.5-fold increase), PALLD (1.9-fold increase), and MAP4K4 (1.6-fold increase) were deregulated in the direction of inhibiting those cellular events when FLRT2 was upregulated. Contrastingly, CXCR4 (2.5-fold increase) and DKK1 (1.9-fold increase) were deregulated in the direction of stimulating the events when FLRT2 was downregulated. All five proteins are known to regulate cell adhesion and movement in various normal and cancer cells^40–42^. Whether FLRT2 itself interacts with collagen I should be established through further experiments.

The interaction partners of FLRT family proteins are known to be either homophilic (FLRT-FLRT) or heterophilic (FLRT-Unc5)^39^. FLRT2-fibronection interaction has also been demonstrated in ATDC5 chondroprogenitor cells^24^. Notably, MDA-MB-231 cells did not display strong adhesion to fibronectin-coated beads in cell culture, a protein that shares a high structural similarity with FLRT2. One should also mention that, when FLRT2 was overexpressed in MDA-MB-231 cells, the growth rate as well as the migration rate was only slightly depressed, in contrast to a larger change when the gene was downregulated. This may indicate that a threshold FLRT2 expression level is not only sufficient but also pivotal in the maintenance of the appropriate cell adhesion and migration.

Taken together, it has been shown in this study that FLRT2 expression is suppressed in breast cancer as a result of hypermethylation at promoter CpG sites, which is related to an unfavorable prognosis in cancer patients. In addition, FLRT2 has been shown to suppress the viability and invasive capacity of breast cancer cells. Finally, a proposal has been made that collagen I interacts with FLRT2. These observations suggest FLRT2 to be a potential tumor suppressor that could be used as an epigenetically regulated marker for the diagnosis of breast cancer. Further studies in developing a proper treatment option to control the FLRT2 expression is expected to improve prognosis.

## Materials and Methods

### Study subjects

All patients provided written informed consent to donate removed tissue to the National Cancer Center (NCC) in Korea and samples were obtained according to protocols approved by the Research Ethics Board of NCC. Twenty breast cancer tissues were obtained from patients who had undergone surgery between 2013 and 2014 at NCC.

### Cell culture and transfection

Normal breast cell line MCF-10A and breast cancer cell lines MCF7, T47D, MDA-MB-231, HCC38, and HCC1395 were purchased from ATCC (Manassas, VA, USA) and cultured under the optimal conditions required according to instructions by ATCC. To over-express FLRT2 in cultured cells, a recombinant expression vector containing the open reading frame was designed and constructed as an OmicsLink ORF Expression Clone (Genecopoeia, Rockville, MD, USA). Pre-designed siRNAs for FLRT2 silencing were purchased from Bioneer (Daejeon, South Korea). The most effective siRNA concentration was determined by transfecting cells with 10, 40, and 80 nM siRNA. All transfection processes were performed with Lipofectamine 2000 (Invitrogen, Carlsbad, CA, USA) by following the supplier’s protocol.

### Real-time reverse transcription (RT)-PCR

Chromosomal DNA and total RNA were extracted with the AllPrep DNA/RNA Mini kit (Qiagen, Valencia, CA, USA), with elution in 100 µl and 30 µl respectively. cDNA was synthesized from 1 µg of total RNA by means of ReverTra Ace qPCR RT Master Mix with gDNA Remover (Toyobo, Osaka, Japan). Gene expression was assessed by real-time RT-PCR. For quantification, one microliter of DNA was used on an ABI 7300 Thermal Cycler (Applied Biosystems, Foster City, CA, USA) with gene-specific primers (Supplementary Table S3). Values were normalized relative to the GAPDH level with the 2^−ΔΔCt^ method. For methylation-specific PCR (MSP), sodium bisulfite modification of genomic DNA was carried out on 0.1 mg of purified DNA using an EpiTect Bisulfite Kit (Qiagen) in accordance with the manufacturer’s instructions. Primers for methylation detection were designed with MethPrimer (http://www.urogene.org/methprimer) (Supplementary Table S3). MSP products were detected with a Power SYBR Green Kit (Applied Biosystems, Foster City, CA, USA).

### Genome-wide expression analysis

Total RNA was extracted from cultured cells and forwarded for array analysis on duplicate chips (Macrogen, Seoul, Korea). In brief, an IlluminaHT-12 v4.0 Expression BeadChip (Illumina Inc., San Diego, CA, USA) with 47,000 probes was detected using biotinylated cRNA in accordance with the manufacturer’s instruction. The array was scanned with an Illumina BeadArray Reader confocal scanner and results were extracted with Illumina GenomeStudio v2011.1. For pathway analysis, genes with a less than 1.5-fold expression change were filtered out, and the remaining genes were analyzed with Ingenuity Pathway Analysis (IPA) software (Qiagen). All array data have been uploaded to the Gene Expression Omnibus (GEO) database, and can be accessed via their website (http://www.ncbi.nlm.nih.gov/geo/; accession number, GSE85247).

### Cell proliferation and colony formation assay

As a cell proliferation assay, cells were transfected and, 24 h later, seeded on a 96-well plate at a concentration of 3,000 cells per well. On the day of examination, 10 µl of CCK-8 solution (Dojindo, Tokyo, Japan) were added to each well and absorbance was measured by following the supplier’s protocol. As a colony formation assay, 5,000–10,000 cells were seeded in a 60 mm culture dish, depending on cell type, and incubated for 10–20 days until distinguishable colony sizes appeared. Colonies were fixed with 70% methanol solution and stained with a 0.01–0.1% crystal violet solution.

### Migration assay

For the migration assay, cells were starved for 24 hrs and then seeded on a 6.5 mm migration chamber with 8.0 µm pores (Corning, Corning, NY, USA) in serum-free medium. Cells that had migrated were fixed with 70% methanol 4–24 hrs after incubation and then stained with crystal violet solution. Cells were examined with a light microscope to count the stained cells in random areas within each chamber. At least four areas in each chamber were captured and numerically analyzed with Image J.

### Analysis of apoptosis by flow cytometry

Cultured cells, including attached and floating cells, were harvested 24 h after transfection. The cells were then suspended with binding buffer to achieve a concentration of 1 × 10^6^ cells/mL and treated with 10 µl of two staining solutions containing FITC Annexin V and Propidium Iodide per 1 × 10^5^ cells in accordance with the protocol of the FITC Annexin V Apoptosis Detection Kit (BD Biosciences, Franklin Lakes, NJ, USA). Samples were analyzed using a FACS Canto II flow cytometer (BD Technologies) and read with a 488-nm laser.

### Adhesion assay

Adhesion assay was performed by means of a colorimetrically formatted CytoSelect 48-Well Cell Adhesion Assay Kit (Cell Biolabs, San Diego, CA, USA) following the supplier’s protocol. Briefly, cultured cells were suspended in serum-free medium at a concentration of 1 × 10^6^ cell/ml. 150 µl of the cell suspension were added into each well and incubated for 60 min in a cell culture environment for adhesion. Non-adherent cells were then washed out 4 times with PBS, and 200 µl of Cell Stain Solution were added to stain adherent cells. After staining at room temperature, the wells were dried in air and 200 µl of Extraction Solution were added to each well. The extracted sample was transferred to a 96-well microtiter plate in order to measure optical density with a plate reader at 560 nm.

### Statistical analysis

The samples that contained the expression and CpG methylation data of breast cancer patients were downloaded from the Cancer Genome Atlas (TCGA, http://cancergenome.nih.gov/). Methylation profiling from breast cancer patients that contained the cancer grade information were obtained from the Gene Expression Omnibus (http://www.ncbi.nlm.nih.gov/geo/). A multiple comparison was applied in the microarray analysis using Benjamini-Hochberg method, as previously described^43^ to avoid false discoveries. Log transformation was applied to the expression array data and quantile normalized. The rate of Distant Metastasis Free Survival (DMSF) was obtained from the GOBO database (http://co.bmc.lu.se/gobo). The samples were further stratified according to the estrogen receptor status, ER− and ER+, and tissue types. Statistical significance of all experimental differences was determined by unpaired Student’s t-test. A *P*-value of less than 0.05 was deemed statistically significant.

## Electronic supplementary material

Supplementary PDF File

## References

[1] Jones PA, Baylin SB (2002). The fundamental role of epigenetic events in cancer. Nat. Rev. Genet..

[2] Gyorffy B (2016). Aberrant DNA methylation impacts gene expression and prognosis in breast cancer subtypes. Int. J. Cancer.

[3] Chuikov S (2004). Regulation of p53 activity through lysine methylation. Nature.

[4] Yamamoto S (2014). JARID1B is a luminal lineage-driving oncogene in breast cancer. Cancer Cell.

[5] Liu L, Xu C, Hsieh JT, Gong J, Xie D (2016). DAB2IP in cancer. Oncotarget.

[6] Li Y, Liang J, Hou P (2015). Hypermethylation in gastric cancer. Clin. Chim. Acta.

[7] Krassenstein R (2004). Detection of breast cancer in nipple aspirate fluid by CpG island hypermethylation. Clin. Cancer. Res..

[8] Schnekenburger, M., Karius, T. & Diederich, M. Regulation of epigenetic traits of the glutathione S-transferase P1 gene: from detoxification toward cancer prevention and diagnosis. *Front. Pharmacol*. **5**, 170 (2014). 10.3389/fphar.2014.00170 PMC4100573 25076909

[9] Saxena A (2012). GSTP1 methylation and polymorphism increase the risk of breast cancer and the effects of diet and lifestyle in breast cancer patients. Exp. Ther. Med.

[10] Zhang J, Babic A (2016). Regulation of the MET oncogene: molecular mechanisms. Carcinogenesis.

[11] Nones K (2014). Genome-wide DNA methylation patterns in pancreatic ductal adenocarcinoma reveal epigenetic deregulation of SLIT-ROBO, ITGA2 and MET signaling. Int. J. Cancer.

[12] Kim SJ, Kim YS, Jang ED, Seo KJ, Kim JS (2015). Prognostic Impact and Clinicopathological Correlation of CD133 and ALDH1 Expression in Invasive Breast Cancer. J. Breast Cancer.

[13] Sebova K (2011). RASSF1A and CDH1 hypermethylation as potential epimarkers in breast cancer. Cancer Biomark..

[14] Seniski, G.G. *et al.* ADAM33 gene silencing by promoter hypermethylation as a molecular marker in breast invasive lobular carcinoma. *BMC Cancer* **9**, 80 (2009). 10.1186/1471-2407-9-80 PMC2660367 19267929

[15] Kang S (2013). Stage-specific methylome screen identifies that NEFL is downregulated by promoter hypermethylation in breast cancer. Int. J. Oncol..

[16] Lacy SE, Bonnemann CG, Buzney EA, Kunkel LM (1999). Identification of FLRT1, FLRT2, and FLRT3: a novel family of transmembrane leucine-rich repeat proteins. Genomics.

[17] Akita T, Kumada T, Yoshihara S, Egea J, Yamagishi S (2016). Ion channels, guidance molecules, intracellular signaling and transcription factors regulating nervous and vascular system development. J. Physiol. Sci..

[18] Lu YC (2015). Structural Basis of Latrophilin-FLRT-UNC5 Interaction in Cell Adhesion. Structure.

[19] Seiradake E (2014). FLRT structure: balancing repulsion and cell adhesion in cortical and vascular development. Neuron.

[20] Kobe B, Kajava AV (2001). The leucine-rich repeat as a protein recognition motif. Curr. Opin. Struct. Biol.

[21] Evdokimov AG, Anderson DE, Routzahn KM, Waugh DS (2001). Unusual molecular architecture of the Yersinia pestis cytotoxin YopM: a leucine-rich repeat protein with the shortest repeating unit. J. Mol. Biol..

[22] Price SR, Evans PR, Nagai K (1998). Crystal structure of the spliceosomal U2B″-U2A′ protein complex bound to a fragment of U2 small nuclear RNA. Nature.

[23] Haines BP, Wheldon LM, Summerbell D, Heath JK, Rigby PW (2006). Regulated expression of FLRT genes implies a functional role in the regulation of FGF signalling during mouse development. Dev. Biol..

[24] Flintoff KA, Arudchelvan Y, Gong SG (2014). FLRT2 interacts with fibronectin in the ATDC5 chondroprogenitor cells. J. Cell. Physiol..

[25] Pangeni, R.P. *et al.* The GALNT9, BNC1 and CCDC8 genes are frequently epigenetically dysregulated in breast tumours that metastasise to the brain. *Clin. Epigenetics* **7**, 57 (2015). 10.1186/s13148-015-0089-x PMC4457099 26052355

[26] Hibi K (2012). FBN2 methylation is detected in the serum of colorectal cancer patients with hepatic metastasis. Anticancer Res..

[27] Tai, Y.L. *et al.* An EGFR/Src-dependent beta4 integrin/FAK complex contributes to malignancy of breast cancer. *Sci. Rep.* **5**, 16408 (2015). 10.1038/srep16408 PMC4637903 26549523

[28] Wu MX, Ao Z, Prasad KV, Wu R, Schlossman SF (1998). IEX-1L, an apoptosis inhibitor involved in NF-kappaB-mediated cell survival. Science.

[29] Jiang W (2011). S100B promotes the proliferation, migration and invasion of specific brain metastatic lung adenocarcinoma cell line. Cell Biochem. Funct..

[30] Al-Rawi MA, Rmali K, Mansel RE, Jiang WG (2004). Interleukin 7 induces the growth of breast cancer cells through a wortmannin-sensitive pathway. Br. J. Surg..

[31] Cardones AR, Murakami T, Hwang ST (2003). CXCR4 enhances adhesion of B16 tumor cells to endothelial cells *in vitro* and *in vivo* via beta(1) integrin. Cancer Res..

[32] Ferdous Z (2010). A role for decorin in controlling proliferation, adhesion, and migration of murine embryonic fibroblasts. J. Biomed. Mater. Res. A.

[33] Chen L, Li M, Li Q, Wang CJ, Xie SQ (2013). DKK1 promotes hepatocellular carcinoma cell migration and invasion through beta-catenin/MMP7 signaling pathway. Mol. Cancer.

[34] Yang ZY (2013). Metallopanstimulin-1 regulates invasion and migration of gastric cancer cells partially through integrin beta4. Carcinogenesis.

[35] Kwiatkowska E (2016). Effect of 3-bromopyruvate acid on the redox equilibrium in non-invasive MCF-7 and invasive MDA-MB-231 breast cancer cells. J. Bioenerg. Biomembr..

[36] Shintani Y, Hollingsworth MA, Wheelock MJ, Johnson KR (2006). Collagen I promotes metastasis in pancreatic cancer by activating c-Jun NH(2)-terminal kinase 1 and up-regulating N-cadherin expression. Cancer Res..

[37] Wu Y (2016). Methylation profiling identified novel differentially methylated markers including OPCML and FLRT2 in prostate cancer. Epigenetics.

[38] Ordway JM (2007). Identification of novel high-frequency DNA methylation changes in breast cancer. PLoS One.

[39] Yamagishi S (2011). FLRT2 and FLRT3 act as repulsive guidance cues for Unc5-positive neurons. EMBO J.

[40] Barber AG (2015). PI3K/AKT pathway regulates E-cadherin and Desmoglein 2 in aggressive prostate cancer. Cancer Med.

[41] Ghiorzo P (2012). CDKN2A is the main susceptibility gene in Italian pancreatic cancer families. J. Med. Genet..

[42] Santhana Kumar K (2015). The Ser/Thr kinase MAP4K4 drives c-Met-induced motility and invasiveness in a cell-based model of SHH medulloblastoma. Springerplus.

[43] Benjamini Y, Hochberg Y (1995). Controlling the False Discovery Rate: A Practical and Powerful Approach to Multiple Testing. Journal of the Royal Statistical Society. Series B (Methodological).

