# Supplementary material for: Epigenetically regulated Fibronectin leucine rich transmembrane protein 2 (FLRT2) shows tumor suppressor activity in breast cancer cells
Source: Sci Rep. 2017 Mar 21;7:272. doi: 10.1038/s41598-017-00424-0 (PMC5428463; doi:10.1038/s41598-017-00424-0)

Epigenetically regulated Fibronectin leucine rich transmembrane protein 2 (FLRT2) shows tumor suppressor activity in breast cancer cells

Hansol Bae, Byungtak Kim, Hyunkyung Lee, Seungyeon Lee, Han-Sung Kang, and Sun Jung Kim

Supporting Information

Table S1. Public methylation microarray data of breast tissues adopted in this study

**Normal (N=33)**		
GSM801711, GSM801719, GSM801720, GSM801721, GSM801722, GSM801742, GSM801747, GSM801754, GSM801758, GSM801772, GSM801789, GSM801795, GSM801815, GSM801816, GSM801817, GSM801821, GSM801822, GSM801824, GSM801827, GSM801832, GSM801834, GSM819045, GSM819047, GSM819049, GSM819051, GSM553865, GSM553866, GSM553867, GSM553868, GSM553869, GSM553870, GSM553871, GSM553872		
**Cancer (N=112)**		
**Grade I (N=34)**	**ER+**	GSM801706, GSM801707, GSM801708, GSM801709, GSM801713, GSM801723, GSM801724, GSM801727, GSM801733, GSM801737, GSM801739, GSM801745, GSM801749, GSM801752, GSM801755, GSM801759, GSM801760, GSM801762, GSM801773, GSM801775, GSM801776, GSM801781, GSM801794, GSM801810, GSM801811, GSM801829, GSM801838, GSM801839, GSM80184
	**ER-**	GSM801734, GSM801753, GSM801766, GSM801768, GSM801771
**Grade II (N=59)**	**ER+**	GSM801712, GSM801714, GSM801715, GSM801726, GSM801730, GSM801732, GSM801736, GSM801738, GSM801741,GSM801743, GSM801746, GSM801756, GSM801761, GSM801763, GSM801764, GSM801769, GSM801770, GSM801777, GSM801779, GSM801782, GSM801786, GSM801788,GSM801790, GSM801791, GSM801798, GSM801800, GSM801803,GSM801804, GSM801805, GSM801807, GSM801812, GSM801813, GSM801818, GSM801819, GSM801823, GSM801825, GSM801826, GSM801828, GSM801830,GSM801835
	**ER-**	GSM801725, GSM801728, GSM801729, GSM801746, GSM801748, GSM801774, GSM801780, GSM801783, GSM801785, GSM801787, GSM801792, GSM801796, GSM801797, GSM801799, GSM801801, GSM801808, GSM801809, GSM801814, GSM801836, GSM801837
**Grade III (N=19)**	**ER+**	GSM801717, GSM801765, GSM801802, GSM801820, GSM801833, GSM801842
	**ER-**	GSM801710, GSM801716, GSM801735, GSM801740, GSM801744, GSM801751, GSM801757, GSM801767, GSM801778, GSM801784, GSM801806, GSM801831, GSM801841

Table S2. Public expression microarray data of breast cancer cell lines adopted in this study

Cell type	GEO database ID
MCF-10A	GSM1017484, GSM1017485, GSM1017486
MDA-MB-231	GSM1017490, GSM1017491, GSM1017492
T-47D	GSM1017511, GSM1017512, GSM1017513
MCF-7	GSM1017487, GSM1017488, GSM1017489

Table S3. Sequences of primers applied in this study

Genes	Forward primer (5'→3')	Reverse primer (5'→3')
**MSP**[a]		
FLRT2 M	TCGTTGGTAATTTTAATAAATTCGG	AAACGAAAACTTTATTCACGTACGT
FLRT2 U	TTGTTGGTAATTTTAATAAATTTGG	AACAAAAACTTTATTCACATACATT
**Real-time RT-PCR**		
GAPDH	CAGGAGGCATTGCTGATGAT	GAAGGCTGGGGCTCATTT
FLRT2	ACCCTTGGTTTTGTGACTGC	AGGACCTTGGCACATGAAAC
SFRP1	CAAGCCCCAAGGCACAAC	TCATCCTCAGTGCAAACTCG
CCND2	CACCTGGATGCTGGAGGTC	GAGGCCAGGAACATGCAGA
FBN2	TGGACTAACAATTCTGAACCAGAC	CAACATCTATACAATCTCCATTTGC
BCAN	CGGATCAGACCGTGAGGTAT	GTTCTCCATTTAGGTCTTCAGCA
PLSCR4	TACCAGGACCCCCTGGAAC	GGCTGGTACAAAGGGAAGGT
GIPC2	GGTCAAAGGCTGGAAAGTCA	TTTCAGAAGGCATTTCTTCCA
LCAT	TACTATCACTGGGCGCTGTC	GTCACCCCACTCCTTCTCAG
APCDD1	CAGGCTGTGAAGTAAGGTCAGG	TTGCTGCCATAATAAAATTGG
FBN1	TGGGGAGTGTATTGATGTTGA	AATGTCTCGGCATTCTGTCC
PLD5	AGCACAAGCACCAAAAGGAC	GCAGTTGGCTATTTCAGTGC

[a]M, methylated sequence; U, unmethylated sequence.

Figure S1. RT-PCR analysis of FLRT2 in normal and cancerous tissue of the breast. Expression of FLRT2 was examined by real-time RT-PCR for 20 pairs of tumor tissue and nearby normal tissue. Each sample was analyzed twice, and average relative expression levels were plotted.

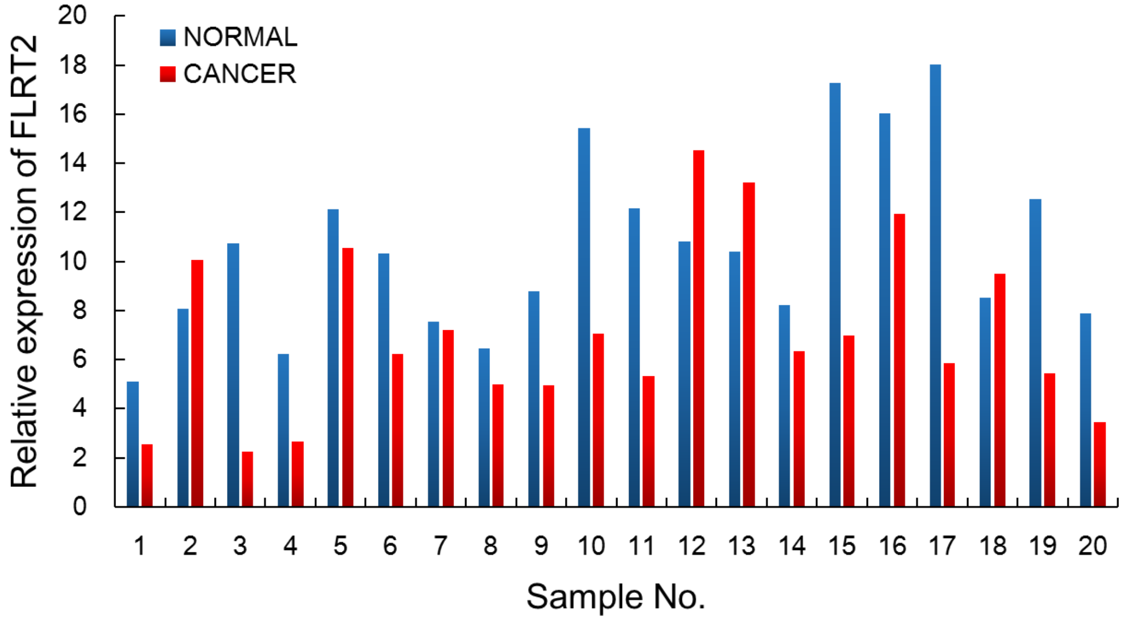

Figure S2. Kaplan-Meier survival analysis of FLRT2 expression in breast cancer patients. Samples from GOBO database (http://co.bmc.lu.se/gobo) were stratified into tertiles based on FLRT2 expression level. The log-rank test was performed in all tumor samples, as well as in different tumor subtypes, using distant metastasis-free survival (DMFS) as the endpoint. Higher FLRT2 expression is significantly associated with increased DMFS in all tumors, ER+, luminal A, and lymph node negative tissue.

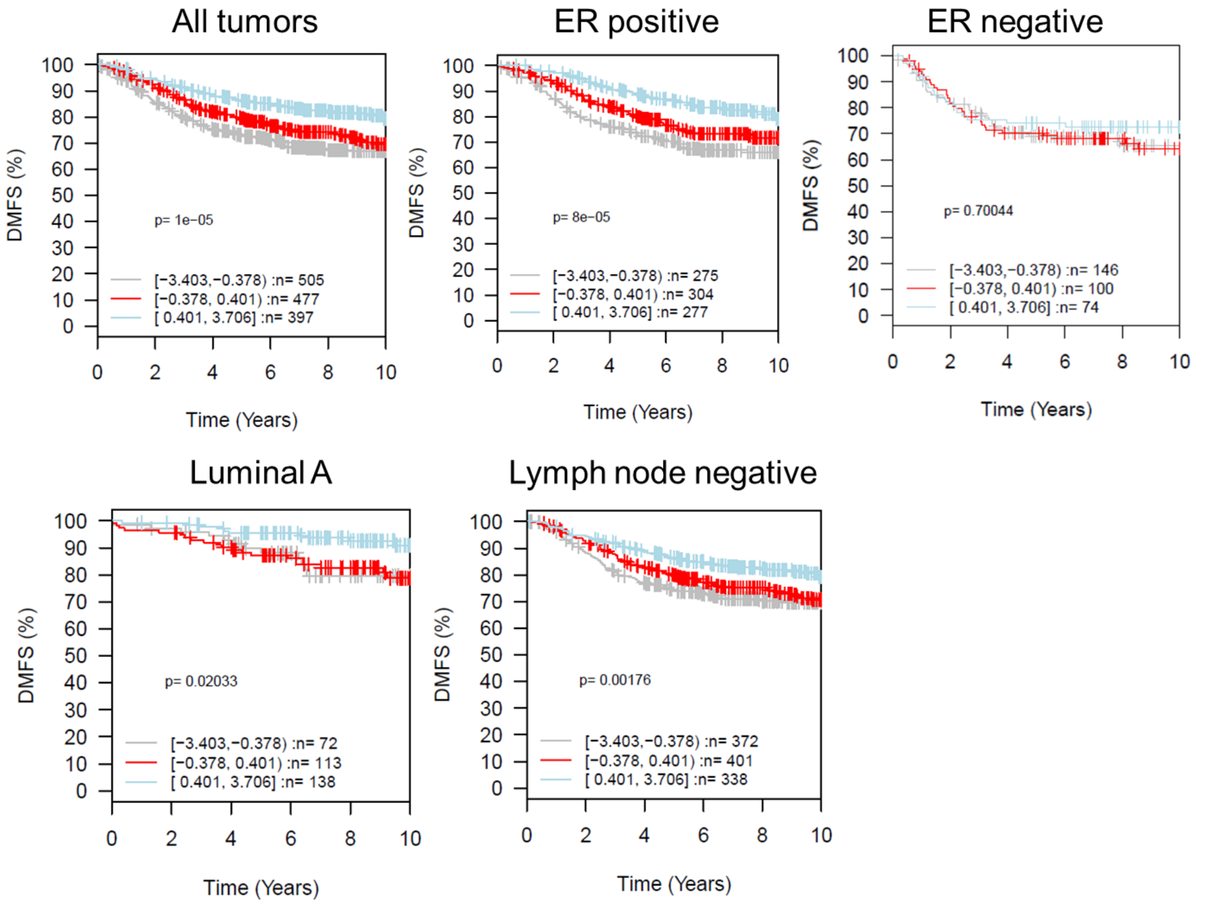

Figure S3. RT-PCR analysis of FLRT2 after inducing downregulation and overexpression in cell lines. Overexpression (A) and downregulation (B) of FLRT2 was induced in MCF-7 and MCF-10A, respectively, and assessed by real-time RT-PCR. All experiments were performed at least three times, and the results are shown as the mean with standard errors. Control: control plasmid vector in A and control siRNA in B.

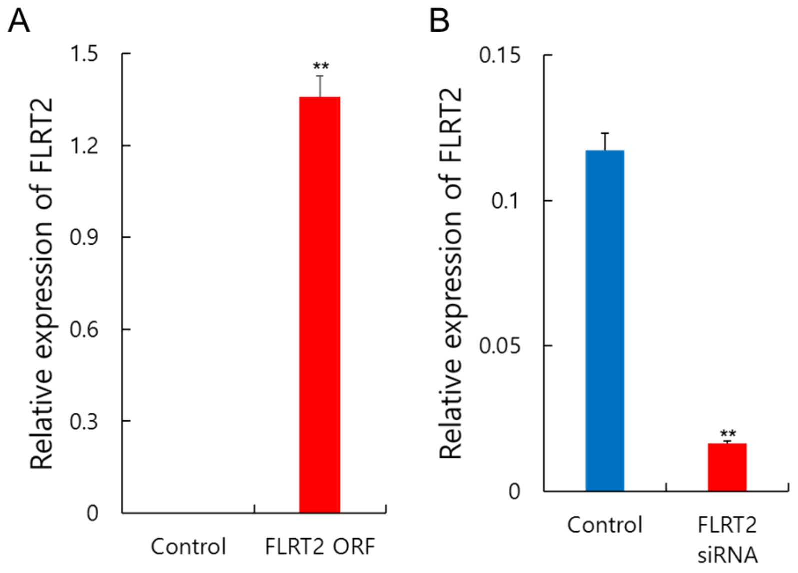

Supplement: Supplementary file 1 — Supplementary PDF File [file 41598_2017_424_MOESM1_ESM.pdf]
